# Molecular Characterisation of a Supergene Conditioning Super-High Vitamin C in Kiwifruit Hybrids

**DOI:** 10.3390/plants8070237

**Published:** 2019-07-22

**Authors:** John McCallum, William Laing, Sean Bulley, Susan Thomson, Andrew Catanach, Martin Shaw, Mareike Knaebel, Jibran Tahir, Simon Deroles, Gail Timmerman-Vaughan, Ross Crowhurst, Elena Hilario, Matthew Chisnall, Robyn Lee, Richard Macknight, Alan Seal

**Affiliations:** 1 New Cultivar Innovation, The New Zealand Institute for Plant & Food Research Limited, Private Bag 4704, Christchurch 8140, New Zealand; 2 Biochemistry Department, University of Otago, Dunedin 9054, New Zealand; 3 New Cultivar Innovation, The New Zealand Institute for Plant & Food Research Limited, Private Bag 11600, Palmerston North 4442, New Zealand; 4 New Cultivar Innovation, The New Zealand Institute for Plant & Food Research Limited, Private Bag 92169, Auckland Mail Centre, Auckland 1142, New Zealand; 5 New Cultivar Innovation, The New Zealand Institute for Plant & Food Research Limited, 412 No 1 Road, RD 2 Te Puke 3182, New Zealand

**Keywords:** kiwifruit, genomics, polyploidy, breeding, ascorbic acid, vitamin C

## Abstract

During analysis of kiwifruit derived from hybrids between the high vitamin C (ascorbic acid; AsA) species *Actinidia eriantha* and *A. chinensis*, we observed bimodal segregation of fruit AsA concentration suggesting major gene segregation. To test this hypothesis, we performed whole-genome sequencing on pools of hybrid genotypes with either high or low AsA fruit. Pool-GWAS (genome-wide association study) revealed a single Quantitative Trait Locus (QTL) spanning more than 5 Mbp on chromosome 26, which we denote as qAsA26.1. A co-dominant PCR marker was used to validate this association in four diploid (*A. chinensis* × *A. eriantha*) × *A. chinensis* backcross families, showing that the *A. eriantha* allele at this locus increases fruit AsA levels by 250 mg/100 g fresh weight. Inspection of genome composition and recombination in other *A. chinensis* genetic maps confirmed that the qAsA26.1 region bears hallmarks of suppressed recombination. The molecular fingerprint of this locus was examined in leaves of backcross validation families by RNA sequencing (RNASEQ). This confirmed strong allelic expression bias across this region as well as differential expression of transcripts on other chromosomes. This evidence suggests that the region harbouring qAsA26.1 constitutes a supergene, which may condition multiple pleiotropic effects on metabolism.

## 1. Introduction

Kiwifruit cultivars of *Actinidia chinensis* are known as a rich source of dietary vitamin C (AsA). However, the related species *A. eriantha* has AsA concentrations in its fruit of up to 800 mg/100 g fresh weight but has small fruit with a bland flavour [1]. Recently a large-fruited high AsA *A. eriantha* cultivar (‘White’) has been described [2]. If this high concentration could be transferred by crossing to more palatable kiwifruit species, an ultra-high health fruit could be developed. The availability of high-quality genome sequences for *A. eriantha* [3] as well as *A. chinensis* var. *chinensis* [4,5] provides the basis for functional and genetic approaches to aid such introgression.

The dominant pathway of AsA biosynthesis in *Actinidia* species including *A. eriantha* appears to be the L-galactose pathway [1], with AsA biosynthesis occurring early in fruit development, and then declining. The control of this pathway lies in an early committed step of biosynthesis in the enzymes GDP-galactose phosphorylase (GGP) and GDP-mannose epimerase (GME), with some input from GDP mannose pyrophosphorylase (GMP) [6,7]. Transformation of plants to over-express GGP results in a several fold increase in fruit or tuber ascorbate [8] and over-expression of GME, which by itself has little effect, synergistically increases ascorbate yet further [9]. Oxidised ascorbate is also reduced by several enzymes which have also been implicated in controlling ascorbate concentrations, as have a range of transcription factors and other regulators [7]. In addition, the upstream open reading frame of the GGP gene has a role in controlling translation of the GGP gene and thus ascorbate concentration, forming a feed-back control loop in response to elevated ascorbate [9]. Thus, a complex of enzymes and regulators controls ascorbate concentration in plants, any of which may explain why *A. eriantha* has such a high ascorbate concentration. 

Both in apples [10] and tomatoes [11] QTL mapping has successfully identified candidate genes for regulation of ascorbate content. In this paper we analyse the genetic basis for why *A. eriantha* has such high ascorbate by studying crosses between *A. eriantha* and other *Actinidia* species, and locate the chromosomal region conditioning super-high ascorbate levels in *A. eriantha*.

## 2. Results

### 2.1. Pooled Whole-Genome Sequencing and Genome-Wide Association Study (GWAS)

**Figure 1:**
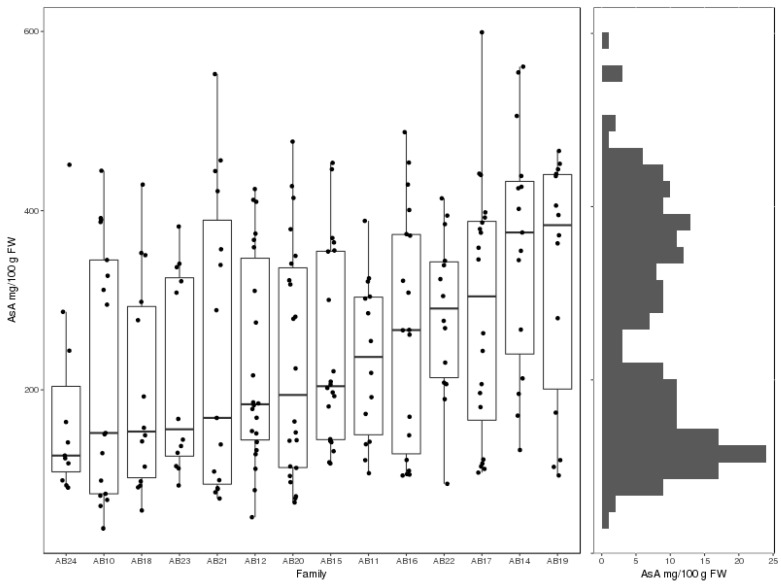
Distributions of vine mean ascorbic acid (AsA) concentration in mg per 100g fresh weight (FW) in tetraploid hybrid *Actinidia* families.

Quantitative HPLC analysis of AsA levels in fruit harvested from tetraploid hybrid *Actinidia* backcross populations revealed evidence for bimodal segregation in all families as well as differences in family medians (Figure 1). 

**Figure 2:**
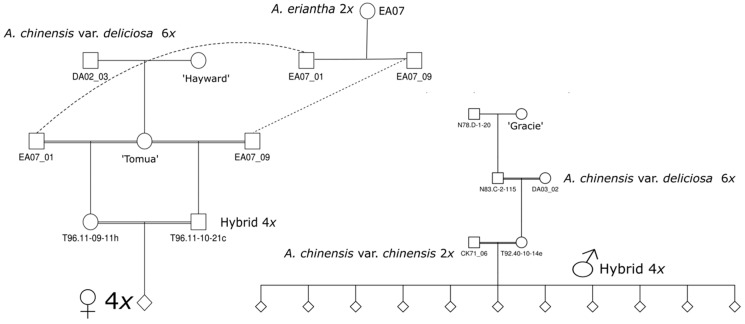
Pedigrees and ploidies of maternal parent (L) and of *A. chinensis* var. *deliciosa* × *A. chinensis* var. *chinensis* males (R) used to generate families used for pooled sequencing.

**Figure 3:**
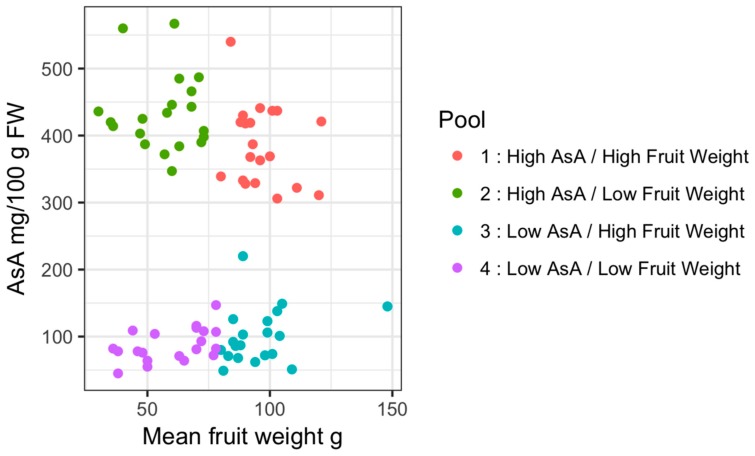
Allocation of samples to sequencing pools.

The parents of these populations were selected from crosses between hexaploid *A. chinensis* var. *deliciosa* and diploid *A. eriantha*, and between hexaploid *A. chinensis* var. *deliciosa* and diploid *A. chinensis* var. *chinensis* (Figure 2). Because of the complex polyploidy genome composition of these populations and the observation of bimodal segregation suggesting a major gene, we conducted genetic analysis by pooled whole-genome sequencing, exploiting the availability of draft genome assemblies of *A. chinensis* var. *chinensis* as reference [4,5]. Since there was wide variation for fruit weight and this was not correlated with AsA levels, we constructed pools by both traits to enable orthogonal, replicated tests of allele frequencies for each trait (Figure 3).

**Figure 4:**
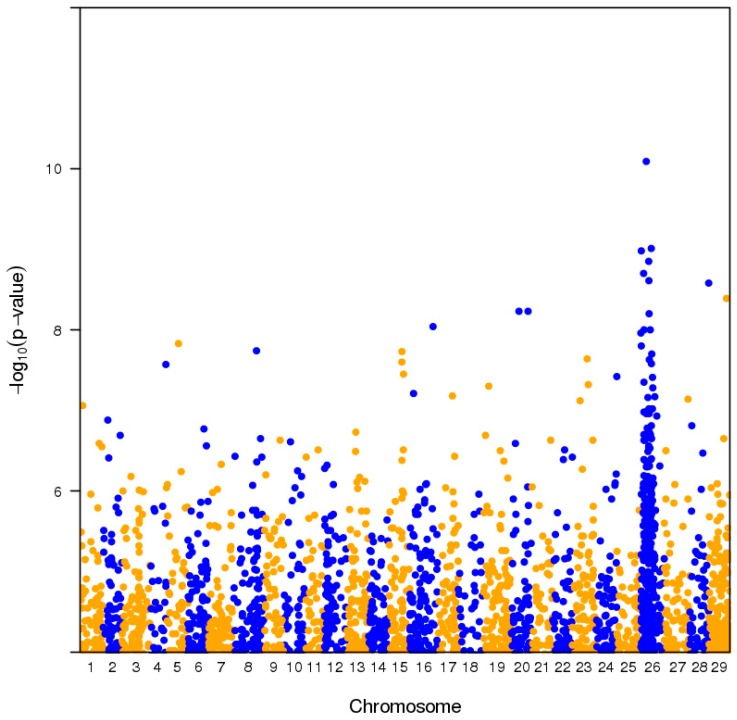
Pool-GWAS (genome-wide association study) scan for fruit AsA concentration level using Popoolation2. Symbols denote significance tests for association of individual SNPs with pool AsA level by Cochran–Mantel–Haenszel (CMH) Chi-Squared Test with normalised allele counts.

**Figure 5:**
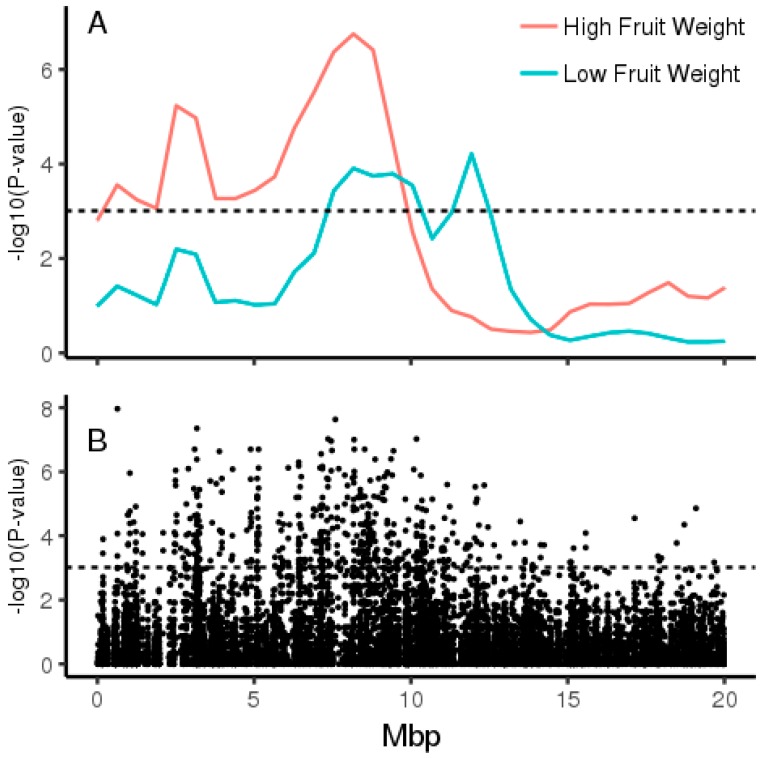
Pool-GWAS analysis of fruit AsA levels on Chromosome 26 using (**A**) 1 Mbp windowed analysis using QTLseqr performed separately in high and low fruit weight pools. (**B**) CMH tests at individual SNPs using Popoolation2. Sites were restricted to fixed variants between *A. eriantha* and *A. chinensis*. Dashed lines denote false discovery rate (FDR) cut-off at *p* < 0.001.

Small insert paired end Illumina sequencing over two lanes yielded 965,452,550 reads with 92.8% Q30, and 86% of reads mapped to the Red5 PS1.1.68.5 pseudomolecules. Pool-GWAS (genome-wide association study) scans performed on both normalised and non-normalised read count data using Popoolation2 [12] revealed a single major QTL for AsA content on Chromosome 26 (Figure 4), but no significant associations with fruit weight (data 4 not shown). Closer inspection of the Chromosome 26 region and windowed analysis using QTLseqR [13] revealed a broad distribution of significant scores for AsA on chromosome 26 (Figure 5; Table A1). Single nucleotide polymorphisms (SNPs) showing association with pool AsA were observed over an interval of 7 Mbp. We denote this major QTL as qAsA26.1.

### 2.2. Validation in Diploid Backcross Populations

To validate this association, we designed a set of high-resolution melting (HRM) assays (Table 3) of the associated variants on chromosome 26 which were homozygous in the low AsA pools (Table A1). Marker KCH00062 targeting the polymorphisms at 7647158-7647167 bp, exhibited agreement with pool AsA levels in 78/80 samples used to construct sequencing pools. A two-way ANOVA model of fruit AsA concentration showed that marker dosage and paternal family explained 79% (*p* < 2 × 10^−16^) and 10% (1.21 × 10^−7^), respectively, of total variance.

**Figure 6:**
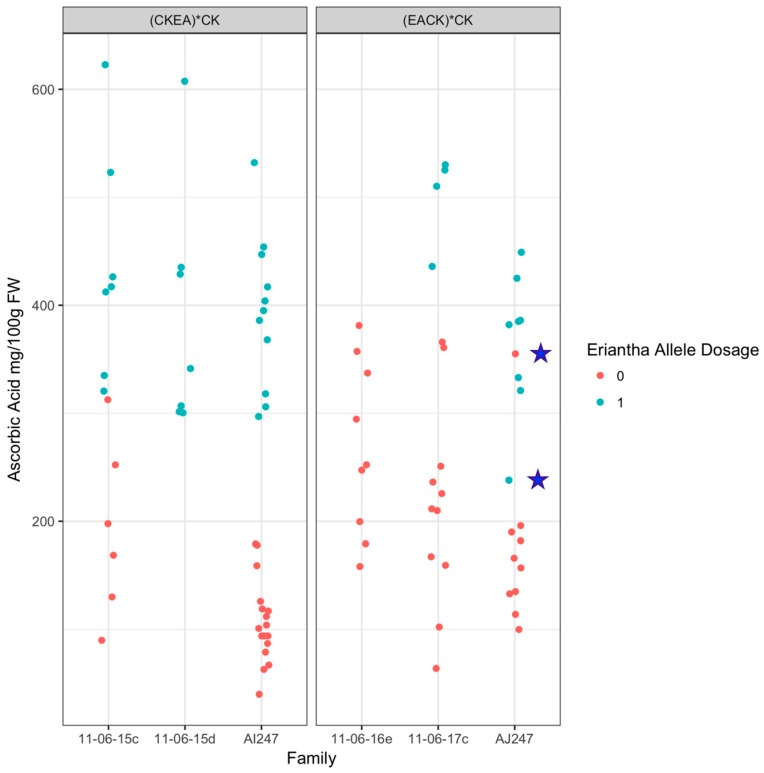
Segregation of the high-resolution melting (HRM) marker KCH00062 in relation to fruit AsA content in six diploid backcross *Actinidia* families. Panels denote back cross type (CKEA*CK (*A. chinensis* × *A. eriantha*) × *A. chinensis*; EACK*CK (*A. eriantha* × *A. chinensis*) × *A. chinensis*). Two putative recombinants are denoted by stars.

This marker was evaluated in a further six diploid backcross families: three (*A. eriantha* × *A. chinensis*) × *A. chinensis* and three (*A. chinensis* × *A. eriantha*) × *A. chinensis* (Figure 6). The maternal parent 11-06-16e of the EACK2 family used by Fraser et al. [14] was homozygous for the *A. chinensis* var. *chinensis* allele and the family did not have any high AsA (>400 mg/100 g FW) fruit. In the AI247 and AJ247 families, ANOVA analysis indicated that the marker explained 78% of the phenotypic variance and residual analysis revealed 3/196 (1.5%) recombinants. The presence of the *A. eriantha* allele is associated with an increase in AsA content of approximately 250 mg/100 g FW. 

Additional HRM markers were evaluated from targets in the 8.2–8.5 Mb interval and three informative markers were identified targeting SNPs at 8,193,148, 8,453,577 and 8,874,229 bp. The marker at 8,453,577 bp exhibited 10% recombination in the tetraploid families but the others exhibited complex segregation patterns and could not be scored. Efforts to design further co-dominant HRM markers in the 0–7 Mbp region were unsuccessful, suggesting that other marker types may be better suited to these highly heterozygous polyploid hybrids. 

### 2.3. Genome Architecture of Actinidia Chromosome 26

**Figure 7:**
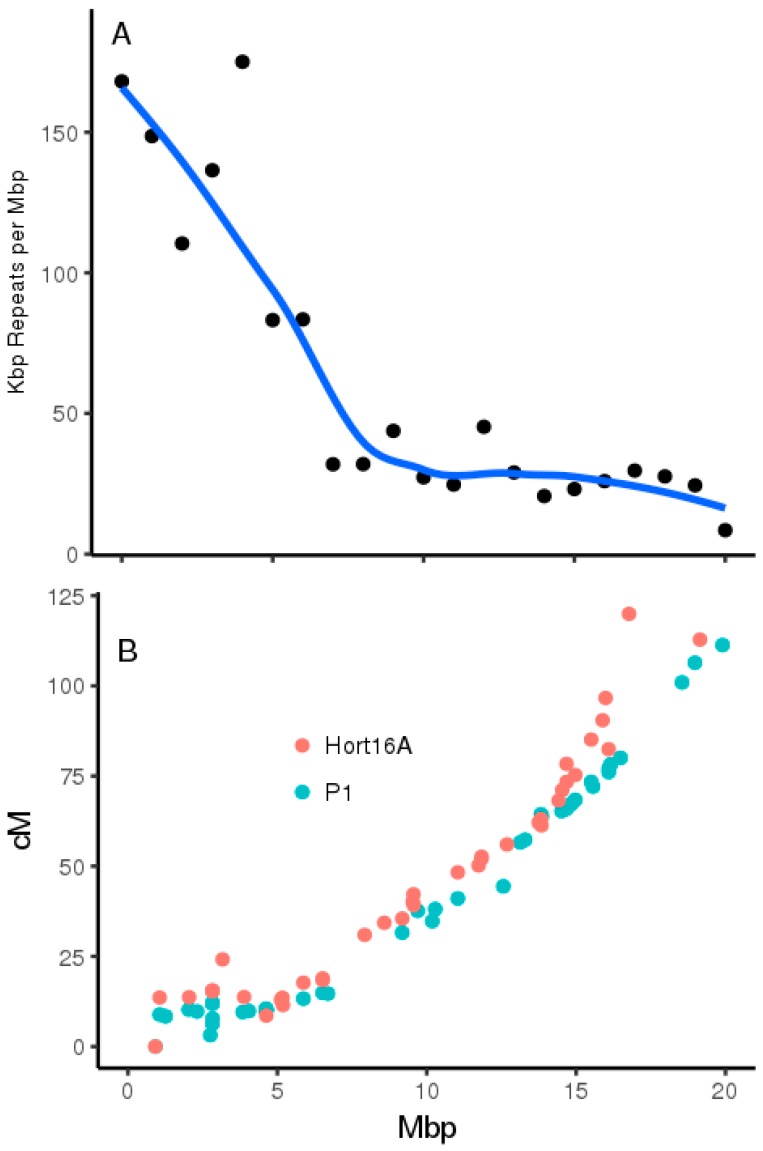
Composition and recombinational landscape of *A. chinensis* chromosome 26. (**A**) Total repeat annotations based on the ‘Red5’ assembly [4] (**B**) Physical versus recombination distance on *A. chinensis* ‘Red5’ Chromosome 26 estimated in male (green) and female (red) parental maps in the ‘Hort 16A × P1’ family [15].

**Figure A1:**
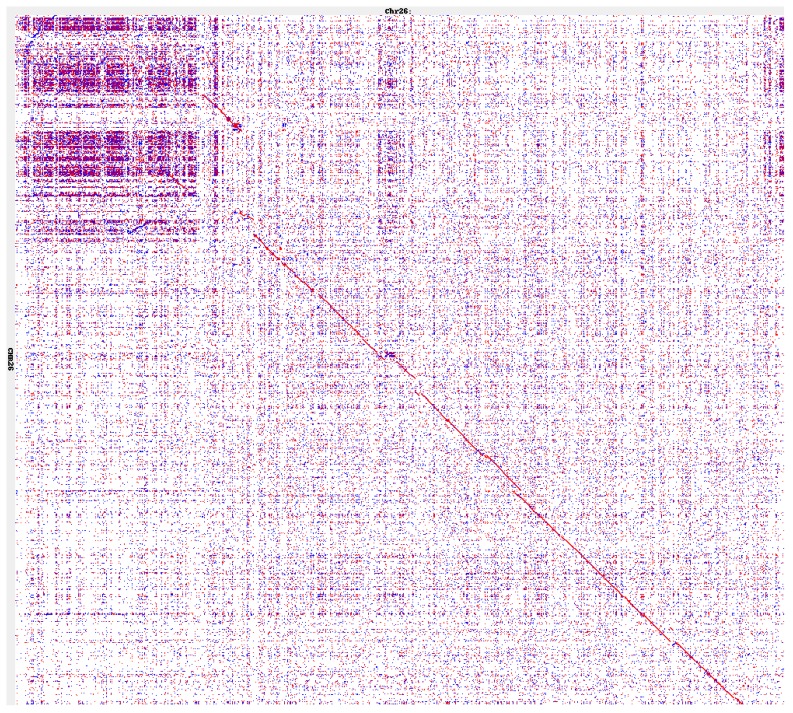
LAST dotplot alignment of chromosome 26 pseudomolecules of *A. chinensis* var. *chinensis* ‘Red5’ (*y*-axis) with *A. eriantha* ‘White’ (*x*-axis).

Inspection of chromosome 26 repeat density and recombination estimates from genetic mapping [15] shows that the location of qAsA26.1 coincides with the boundary of a region with high repeat density and lower recombination (Figure 7). Alignment of the chromosome 26 pseudomolecules from the assemblies of *A. chinensis* ‘Red5’ [4] and the *A. eriantha* ‘White’ [3] indicate that these are highly collinear apart from differences in the terminal repeat-rich region (Figure A1). 

### 2.4. Characterising the qAsA26.1 Introgression in Leaf Tissues

To better characterise the qAsA26.1 introgression we compared the leaf transcriptome and metabolome of low and high AsA progeny in the diploid *A. chinensis* × *A. eriantha* backcross AI247 and AJ247 families used for marker validation. We chose analysis of leaf tissues for ease of reproducible sampling and because it has been shown that *A. eriantha* also exhibits very high leaf AsA levels [1]. Zhang et al. [16] have reported segregation for leaf AsA content in the cross between hexaploid *A. chinensis* var. *deliciosa* and a diploid *A. eriantha* × *A. chinensis* var. *chinensis.* We confirmed by HPLC that leaf AsA levels were higher in samples of immature leaves from backcross progeny carrying the introgression (ACH0007 homozygotes 10.4 mg/100 g FW versus 25.3 mg/100 g FW in heterozygotes; *p* < 0.025 by T-test). These analyses were performed on tissue samples collected in RNALater without the precautions necessary for good preservation of AsA and are therefore lower than previously observed [1]. 

#### 2.4.1. Pooled RNASEQ

**Figure 8:**
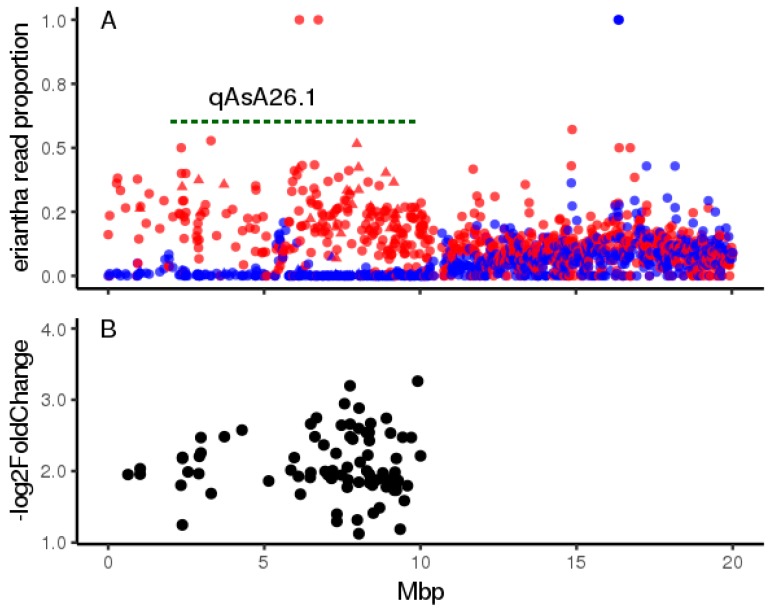
RNASEQ analysis of gene expression on *Actinidia* Chromosome 26. Points denote gene models on the *A. chinensis* ‘Red5’ assembly. (**A**) Allelic expression proportion based on PolyCat read assignment. Red and blue symbols denote high and low AsA pools, respectively. Triangular points denote gene models with transcripts exhibiting differential expression. (**B**) Genomic coordinates and Log2Fold expression differences of transcripts showing significant differential expression.

RNASEQ was performed on three pools of backcross progeny with high fruit AsA which were heterozygous for the introgression and three pools of low AsA progeny lacking it, yielding 21.4–24.4 million reads per library. To determine the patterns of allelic expression on chromosome 26 we performed read assignment using PolyCat [17] based on a set of SNPs identified between *A. chinensis* and *A. eriantha.* This revealed that *A. eriantha* reads were essentially absent in low AsA pools across the first 10 Mbp of Chromosome 26 (Figure 8A), providing additional genetic evidence that recombination is strongly suppressed in this region. 

Differential expression analysis revealed 113 differentially expressed transcripts (DETs) between high and low AsA pools at a false discovery rate (FDR) <0.05 (Supplementary Table S1; Supplementary File 1). Of these, 82 mapped to the qAsA26.1 region of Chromosome 26 (Figure 8B) of these, 61 mapped to annotated gene models. Because of the degree of allelic divergence between the two *Actinidia* species, transcript analysis based on the de novo assembly we used would be expected to frequently reveal novel alleles or splice variants absent in *A. chinensis.*

Prior to library construction, qPCR analysis of individual samples for GGP (GDP-L-galactose phosphorylase), GMD (GDP-D-mannose-4,6-dehydratase), DHAR (dehydro-AsA reductase) showed no evidence for differences between high and low AsA samples (*p* > 0.35 for all T-tests). This observation was confirmed in the RNASEQ data, which showed no evidence of differential expression in transcripts annotated as GGP (KEGG orthology number (KO) K14190), GME (KO K10046), DHAR (KO K08232), VTC4 (K10047) and GMD (K01711) (Supplementary Table S2). 

Significant expression differences were observed for 31 transcripts mapping to chromosomes other than chromosome 26, of which 26 mapped to annotated gene models (Table 1). These include one DET of particular interest. The transcript TRINITY_DN123292_c0_g2_i2 maps to the gene model Acc20170.1 (GenBank: PSS04323.1) encoding a putative GDP-L-fucose synthase 2 homologous to *Arabidopsis* GER2 (KEGG K02377; [18]). We previously reported significantly higher expression of GER in *A. eriantha* compared to *A. chinensis* [1; Figure 4, Panel D] (Authors note: This panel is mis-labelled as GMD). Because fucose synthesis draws upon the same substrate pool as ascorbate [6,7], this may have implications for regulation of mannose channelling to ascorbate. The observation of association between qAsA26.1 genotype and transcript expression at this locus suggests that qAsA26.1 contains transcriptional regulators of carbohydrate metabolism. DETs annotated as beta-glucosidase (Acc03845.1) and pectin acetylesterase (Acc29080.1) were also observed. A further DET of potential functional relevance to AsA metabolism is TRINITY_DN120596_c0_g1_i7 mapping to Acc29025.1 on chromosome 26. This gene is annotated as a component of the dolichol-phosphate mannose synthase complex which mediates mannosylation of glycans [19]. Similar associations between competing carbohydrate metabolic pathway expression and fruit AsA have been reported in studies of tomato interspecific introgressions [20] and ripening [21].

Differential expression was also observed for transcripts homologous to laccase (Acc02955.1) and anthocyanidin reductase (Acc09639.1) mapping to chromosomes 3 and 8, respectively. The differentially expressed transcripts identified on chromosome 26 include both structural genes (Acc29585.1, 4-coumarate CoA ligase; Acc29568.1, Shikimate O-hydroxycinnamoyltransferase) and transcriptional regulators of polyphenol metabolism (Acc18102.1, AtMyb4 homolog). Collectively these observations suggest that polymorphism at qAsA26.1 could exert a direct or indirect influence on polyphenol metabolism. Over-expression of GGP in tomato and strawberry not only increased ascorbate but also increased flavonoids and phenylpropanoids [8]. Further evidence for cross-talk comes from studies of *Arabidopsis vtc* mutants, which have shown that these are also impaired in transcriptional regulation of anthocyanin synthesis [22]. 

#### 2.4.2. Untargetted Metabolomics

Liquid chromatography-MS analysis of leaf extracts from revealed some evidence for more frequent occurrence of elevated levels of flavonoids and phenylpropanoids in those carrying the *eriantha* marker allele (Table 2). This data is from a single time point in an orchard environment and we expect it would be highly influenced by local variability in infection by the pandemic *Pseudomonas syringae* var. Actinidiae [15,23]. Targeted metabolomic analyses of fruit and vine tissues with standards, especially for antioxidant and key carbohydrates is desirable to better characterise the phenotype of qAsA26.1 alleles. 

## 3. Discussion

This study confirms the findings in other horticultural crops such as *Cucumis* [24,25] that pooled sequencing offers a cost-effective and practical means to conduct genome scans in segregating plant populations. Since restricted recombination will preclude further genetic dissection of the locus the application of more sophisticated RNASEQ strategies for analysis of differential transcript usage and QTL [26] would enable a more detailed dissection of allelic and splice variation in future studies. Since our preliminary evidence suggests the potential for complex pleiotropic effects, more detailed metabolic profiling would be desirable. 

The qAsA26.1 QTL is notable for its large effect, size and simple dominant inheritance. Although large effect QTL (>20%) for AsA levels have been reported in other fruits such as apple [27] and tomato [20,21,28], this QTL leads to AsA levels an order of magnitude higher. The structural, linkage and expression data presented here suggest that this QTL constitutes a supergene—a group of tightly linked loci inherited as a single Mendelian locus [29]. Supergenes commonly exert multiple pleiotropic effects and may be key to preserving adaptive variation through protecting a haplotype comprising multiple genes [30]. The qAsA26.1 region bears many similarities to the partially differentiated *Actinidia* sex chromosome (chromosome 25; [31]). Whereas *A. chinensis* is widely distributed in eastern lowland China, *A. eriantha* is restricted to southeastern China [32]. Because AsA can play multiple functional roles in higher plants including as a key redox signal in responses to biotic and abiotic stresses [33], it may be speculated that this extended haplotype has been preserved due to its benefits to adaptive fitness. More detailed functional analysis of the genes lying within qAsA26.1 may permit testing whether the locus action is due to a single as opposed to multiple linked regulators [34] 

The simple inheritance and large effect of this QTL offer some interesting opportunities not only for plant breeding but also for studies of AsA in human and plant physiology. Our findings suggest that practical genetic markers may be easily obtained and applied due to limited recombination and that these could be used to develop breeding lines fixed for high AsA alleles of qAsA26.1. The availability of the ‘White’ genome assembly will greatly simplify design of allele-specific markers that can be applied in highly heterozygous and polyploid backgrounds. Selecting lines with comparable eating qualities expressing ‘normal’ or ‘super-high’ AsA could provide unique materials for human dietary studies. Similarly, the ability to obtain both male and female vines with significantly different AsA content in vegetative tissues would allow replicated testing of hypotheses concerning the role of AsA in plant adaptation and fitness. In addition to marker-based methods, we hope that use of such materials may facilitate discovery of new targets for improvement of AsA levels that are transferable to other crops [35,36]. 

## 4. Materials and Methods 

### 4.1. Plant Materials and Phenotyping

#### 4.1.1. Tetraploid Populations

Pool-GWAS was performed on a set of 80 individuals from 11 (*A. chinensis* var. *deliciosa* × *A. eriantha*) × (*A. chinensis* var. *deliciosa* × *A. chinensis* var. *chinensis*) families (Figure 2). The common maternal parent was a tetraploid high AsA hybrid vine generated by sib mating F_1_ progeny from an *A. chinensis* var. *deliciosa* × *A. eriantha* cross. The *A. eriantha* plants were seedlings originating from seed gifted by the Guangxi Institute of Botany, Guilin, China in 1988. A series of hybrid populations was generated by pollinating this with F_1_ male progeny from an *A. chinensis* var. *deliciosa* × *A*. *chinensis* var. *chinensis* cross. Seedlings were planted at the Plant and Food Research Centre in Kerikeri New Zealand (Lat 35.2 deg S) in 2010 and analysis of fruit AsA was performed in 2013.

#### 4.1.2. Diploid Populations

Marker validation was performed in three (*A. chinensis* var. *chinensis* × *A. eriantha*) × *A. chinensis* var. *chinensis* (CKEA × CK) and three (*A. eriantha* × *A. chinensis* var. *chinensis*) × *A. chinensis* var. *chinensis* (EACK × CK) backcross families, totalling N = 91 individuals. Four of these families (N = 45 plants) were previously used to map petal colour [14]. The two new validation families AJ247 (EACK × CK) and AI247 (CKEA × CK), which were also employed for RNASEQ, had the same *A. eriantha* parentage respectively as populations EACK2 and CKEA3 and CKEA4 reported by Fraser et al. [14].

#### 4.1.3. Ascorbate Analyses

Three whole fruit per seedling were analysed. Each fruit was cut equatorially as a 1 mm slice using a double-bladed knife. The three fruit slices were immediately placed in a plastic 15 mL tube and frozen in liquid nitrogen, then stored at −80 °C until analysis. Fruit were then thawed and centrifuged at 4000× *g* to separate solid material from the juice. It was critical to freeze the fruit before analysis as directly centrifuged fruit juice gave a much lower ascorbate reading. A 0.1 mL aliquot of the juice was then transferred to a micro tube containing 0.9 mL of 0.8% *w*/*v* met phosphoric acid, 2 mM EDTA and 2 mM Tris(2-carboxyethyl)phosphine hydrochloride (TCEP HCL). These samples were then centrifuged at 14,000× *g* for 15 min to clarify the juice and then analysed by HPLC using a rocket column (Altima C18 3 micron from Phenomenex Ltd. (Auckland New Zealand) at 35 °C. Ascorbate was quantified by injecting 5 µL into a Dionex Ultimate^®^ 3000 Rapid Separation LC system (Thermo Scientifc). Instrument control and data analysis was performed using Chromeleon v7.2 (Thermo Scientific). Solvent A was 5 mL methanol, 1mL 0.2M EDTA pH 8.0 and 0.8 mL o-phosphoric acid in 2 L. Solvent B was 100% acetonitrile. The flow was 1.0 mL/min and the linear gradient started with 100% A and B was increased to 30% at 4.5 min, then to 90% B at 6 min. The column was then washed with 100% B and then returned to 100% A. The column was monitored at 245 nm and ascorbate quantified by use of authentic standards. Ascorbate was verified by its UV spectrum. This method gave the sum of oxidised and reduced ascorbate, namely total ascorbate. Ascorbate concentration in the juice was calculated directly and in preliminary assays compared to ascorbate extracted from powdered flesh. The juice method gave about a 5% higher result than the powdered whole fruit method.

### 4.2. Pooled DNA Sequencing

#### 4.2.1. Library Preparation

DNA was isolated from leaf bud tissue collected in spring 2015 using a cetyl trimethylammonium bromide extraction method [37] followed by purification with Qiagen columns and quantitated using the 3500 Genetic Analyzer (Applied Biosystems™, Foster City, CA, USA). Four normalised DNA pools were created of 20 individuals each as shown in Table 3.

Small-insert Thruplex DNA-seq libraries (Rubicon Genomics Ltd.) were synthesised at NZ Genomics Ltd. and sequenced on two lanes of Illumina Hi-Seq 2500 yielding 965 million reads totalling 120 Gbp with 92.8% >Q30. Quality control using FastQC Screen (http://www.bioinformatics.babraham.ac.uk/projects/fastq_screen/) revealed that 85–88% of reads mapped to the Red5 *A. chinensis*_ var. *chinensis* reference version PS1 1.68.5 and 6% mapping to *Actinidia* chloroplast reference [38]. Read data was deposited as Genbank SRA accession PRJNA551536. 

#### 4.2.2. Sequencing Data Processing

Bam alignment files for variant calling were generated following GATK best-practice approaches [39]. Reads were aligned using BWA-MEM v0.7.12 [40] to pseudomolecules of draft assembly version PS1_1.68.5 of *A. chinensis* var. *chinensis* Red5 [4,41], an inbred female genotype related to ‘Hong Yang’ [5]. This draft assembly has been deposited at https://doi.org/10.5281/zenodo.1297303. Bam files were merged using Samtools 1.3.1 [42] and read groups were added using Picard Tools (http://broadinstitute.github.io/picard/) AddOrReplaceReadGroups. Duplicates were marked with Picard MarkDuplicates and indel realignment was performed using GATK RealignerTargetCreator and IndelRealigner. Depth of coverage in regions of interest was calculated using GATK depthofcoverage.

#### 4.2.3. Pooled GWAS and Variant Analysis

Pool-GWAS scans for association of individual SNPs with AsA and fruit weight were performed using Popoolation2 [12]. Variants were summarised using samtools pileup (flags -B -Q 0) and called using popoolation mpileup2sync.jar with option –min-qual 20. Replicated contingency tests were initially performed on non-normalised data over AsA concentration and fruit weight strata using the cmh-test.pl script (flags –min-count 6 –mincoverage 4 –max-coverage 120 --max-coverage 200 --method withreplace). To facilitate comparison over sites with varying coverage, common odd ratios were calculated for significant SNPs using R mantelhaen.test.

Subsequent analyses focused on the genic regions using data resampled with replacement to a read depth of 40 using subsample-synchronised.pl (flags --target-coverage 40). Cochran–Mantel–Haenszel (CMH) tests *p*-values were adjusted for multiple testing using R p.adjust with the Benjamini and Hochberg correction. Output files for CMH tests are available as supplementary material at 10.5281/zenodo.1309045 

To complement the SNP-based analysis, windowed scans for AsA QTL were performed by Next Generation Sequencing Bulked Segregant Analysis (NGS-BSA) [43] using the R package QTLseqr [13]. Input files were generated from VCF files separately for high and low fruit weight samples using samtools bcftools (http://www.htslib.org/doc/bcftools.html), filtering on a set of fixed polymorphisms (file PS1_EA_specific_SNPs.csv.gz in 10.5281/zenodo.3257749) identified between a set of *A. chinensis* genotypes [31] and *A. eriantha* using Bambam intersnp [44]. Two pairs of bulks were compared: High AsA/High Fruit Weight versus Low AsA/High Fruit Weight (pools 1 and 3) and High AsA/Low Fruit Weight and Low AsA/Low Fruit Weight (pools 2 and 4) (Table 2). QTLseqr accepts two population types, F_2_ and RIL. However, the lines used for constructing these pools were the result of backcrosses with the SNP data filtering to collect only alleles segregating in EA, therefore the function simulateAlleleFreq (https://rdrr.io/github/bmansfeld/QTLseqR/man/simulateAlleleFreq.html) was modified to permit analysis of a backcross population type (called BC4x) where the expected allele frequency was 0:0.25 and the expected segregation ratio was 1:1. NGS-BSA analysis was conducted to estimate QTL locations based on allele frequency differences among the pairs of pools. SNPs from all 29 chromosomes were analysed in each single analysis. The population type was set to BC4x, the window size was 1 Mbp, and the simulations were bootstrapped 10,000 times. The FDR was set to *p* < 0.001 based on adjustment by the method of Benjamini and Hochberg [45]. 

For downstream analysis, SNPs and indels were called with the frequentist variant caller Varscan2 v2.4.2 [46], using a hard filter for MAF >0.1, minimum coverage 20 and only reporting sites called in all four pools. VCF files are available at 10.5281/zenodo.1309045.

#### 4.2.4. Chromosomal Analyses

Alignment of Red5 and *A. eriantha* pseudomolecules was performed using Last [47] following repeat masking with Windowmasker [48]. LTR retrotransposons were annotated using LTRHarvest [49]. Recombination distances for chromosome 26 were determined using Joinmap3 (https://www.kyazma.nl/index.php/JoinMap/) from genotyping by sequencing data used to construct a genetic linkage map in the ‘Hort16A × P1’ family (N = 236) [15]. 

#### 4.2.5. PCR Marker Design for Validation

Filtered SNP loci detected by Popoolation2 cmh_test.pl which were homozygous in low AsA pools were used as targets for HRM primer design (Table 4) using the script https://github.com/PlantandFoodResearch/pcr_marker_design/blob/master/design_primers.py [50]. PCR amplification and HRM analysis on a on a Roche LightCycler 480 were performed as described previously [50]. 

### 4.3. RNASEQ and Untargetted Metabolomic Analysis 

#### 4.3.1. Sample Collection and Processing

Tissue was sampled in October 2016 between 11 am and 1 pm from young leaves (3–5 cm) of AI47 and AJ47 families used for marker validation and placed in RNAlater (Sigma-Aldrich Co. LLC, St Louis, MO, USA) for shipping at 4 °C. The first fully expanded leaf from the same vine was also sampled for metabolomic analysis by taking 10 2 mm discs with a biopsy punch and placing into 50% v/v/methanol. Metabolomic analysis is described in Appendix B. RNA was prepared using the Spectrum Plant Total RNA Kit (Sigma-Aldrich Co. LLC, St Louis, MO, USA) and purified with the RNeasy Plant Mini Kit (Qiagen N.V., Hilden, Germany). Poly(A) RNA was isolated from 1.5 µg total RNA using NEXTflex Poly(A) Beads (PerkinElmer, Inc.). Six libraries (three high AsA, three low AsA) were made using the NEXTflex Rapid Directional qRNA-Seq Kit (PerkinElmer, Inc.). Samples were pooled by family and by whether they had AsA phenotype and were heterozygous for KCH0062 marker. Pools were formed as follows: Pool 1 N = 3 AI247 high AsA; Pool 2 AI247 N = 3 low AsA; Pool 3 AJ247 N = 8 high AsA; Pool 4 AJ247 N = 13 low AsA; Pool 5 AJ247 N = 7 high AsA; Pool 6 AJ247 N = 10 low AsA. 

Synthesis of cDNA and quantitative PCR for genes GGP, GMD, T2 and DHAR2 were performed on individual samples from pools 1, 2, 5 and 6 against PP2A catalyst control as reported previously [1]. 

RNA pools were sequenced on the Illumina HiSeq 2500 platform by Otago Genomics Facility (Dunedin, New Zealand) yielding 2.7–3.1 Mbp per library with Q30 <89%. Merged reads were filtered for ribosomal RNA content using SortMeRNA [51], de-interleaved and then trimmed using Trimmomatic [52] with options ILLUMINACLIP:2:30:10 SLIDINGWINDOW:5:20 MINLEN:40 HEADCROP:9. 

#### 4.3.2. RNASEQ Read Assignment 

Unguided alignment to the Red5 version PS1_1.68.5 reference genome was performed using HiSat2 [53]. Alignments were split into species-specific bam files by read assignment with PolyCat [19] based on a homeo-SNP index built from the set of fixed *A.chinensis-A.eriantha* polymorphisms (10.5281/zenodo.3257749 file PS1_EA_specific_SNPs.csv.gz) using the script snpMerge.pl. (https://gist.github.com/jaudall/de14e367b208ccbe3b3be1465167b39b). Bambam counter [44] was used to count reads from the split bam files in Red5 gene models.

#### 4.3.3. RNASEQ Transcript Analysis

A de novo assembly was performed on trimmed reads using Trinity v2.32 [54] yielding an assembly of 345,495 transcripts in 213,327 genes with contig N50 of 798 bp. Transcript abundance was estimated using RSEM [55] and differential expression analysis was performed using DESeq2 Release 3.9 [56]. Transcripts exhibiting differential expression at FDR <0.01 were aligned to the Red5 genome assembly using gmap [57] and intersection with annotated gene models was performed using bedtools [58]. Putative open reading frames and deduced peptides were identified with Transdecoder (https://github.com/TransDecoder) and annotated using GhostKoala [59].

## Figures and Tables

**Table 1:** Genome locations and associated annotations of de novo assembled transcripts not mapping to kiwifruit chromosome 26 which exhibited differential expression between high and low AsA pools at FDR *p* < 0.05.

Contig	Log2Fold Change	Adjusted *p*-Value	Normalised Read Count High Pool Rep 1	Normalised Read Count High Pool Rep 2	Normalised Read Count High Pool Rep 3	Normalised Read Count Low Pool Rep 1	Normalised Read Count Low Pool Rep 2	Normalised Read Count Low Pool Rep 3	Chromosome	Gene Model	Annotation
**TRINITY_DN125630_c0_g2_i1**	−1.6	0.0	197.71	344.66	225.3	70.25	67.17	76.34	CHR3	Acc2955.1	Laccase-7, Precursor (putative)
**TRINITY_DN125630_c0_g5_i2**	−1.5	0.0	260.83	333.18	217.43	74.26	103.72	69.07	CHR3	Acc2955.1	Laccase-7, Precursor (putative)
**TRINITY_DN125630_c0_g5_i3**	−1.33	0.01	162.44	199.49	172.18	49.17	76.06	59.08	CHR3	Acc2955.1	Laccase-7, Precursor (putative)
**TRINITY_DN129828_c1_g5_i5**	1.77	0.02	25.06	4.18	8.85	65.23	70.14	117.24	CHR3	Acc3372.1	T-complex protein 1 subunit epsilon (TCP-1-epsilon) (putative)
**TRINITY_DN118492_c0_g1_i4**	1.87	0.0	14.85	83.55	42.31	334.19	309.19	179.05	CHR3	Acc3845.1	Probable beta-glucosidase btgE, Precursor
**TRINITY_DN118884_c0_g7_i1**	1.87	0.01	1.86	12.53	10.82	90.32	50.38	55.44	CHR3	Acc3860.1	Cytochrome c1 2, heme protein, mitochondrial (Cytochrome c-1 2), Precursor (putative)
**TRINITY_DN129466_c0_g6_i6**	2.12	1.86 × 10^−5^	45.48	250.66	124.95	1171.16	1034.26	664.38	CHR3	Acc3864.1	Magnesium-protoporphyrin IX monomethyl ester (oxidative) cyclase, chloroplastic (Mg)
**TRINITY_DN117715_c1_g3_i1**	1.43	0.02	174.51	378.09	266.63	1407.0	832.74	500.78	CHR3	Acc3890.1	Tubulin beta-4 chain
**TRINITY_DN129466_c0_g6_i3**	1.9	0.0	65.9	237.09	94.45	1040.7	832.74	370.82	CHR5	Acc5209.1	Magnesium-protoporphyrin IX monomethyl ester (oxidative) cyclase, chloroplastic (Mg)
**TRINITY_DN102173_c0_g2_i1**	1.79	0.01	36.2	22.98	25.58	350.24	108.66	65.44	CHR6	Acc12049.1	Polyadenylate-binding protein 8 (PABP-8) (putative)
**TRINITY_DN129080_c0_g2_i1**	−1.17	0.04	426.98	416.73	234.16	141.5	148.17	139.06	CHR8	Acc9639.1	Anthocyanidin reductase ((2S)-flavan-3-ol-forming) (VvANR) (putative)
**TRINITY_DN121164_c0_g1_i4**	−1.56	0.01	297.03	318.55	188.9	65.23	37.54	108.15	CHR9	Acc10699.1	Germin-like protein 5-1, Precursor (putative)
**TRINITY_DN130940_c2_g1_i1**	−2.87	9.73 × 10^−9^	125.31	80.42	61.0	5.02	5.93	1.82	CHR12	Acc29528.1	Mitochondrial import inner membrane translocase subunit TIM17-2 (similar to)
**TRINITY_DN126502_c0_g1_i1**	2.33	1.86 × 10^−5^	4.64	27.16	30.5	159.57	401.06	134.51	CHR13	Acc14777.1	Calcium uniporter protein 6, mitochondrial, Precursor (similar to)
**TRINITY_DN123616_c1_g1_i1**	−2.33	8.05 × 10^−6^	73.33	69.98	72.81	3.01	9.88	8.18	CHR16	Acc18424.1	CUB and EGF-like domain-containing protein 1
**TRINITY_DN126733_c0_g1_i7**	−1.12	0.03	325.8	245.44	301.06	122.44	97.79	144.51	CHR18	Acc20147.1	Zinc finger protein CONSTANS-LIKE 5 (probable)
**TRINITY_DN123292_c0_g2_i2**	−1.65	0.0	270.11	115.93	124.95	37.13	37.54	47.26	CHR18	Acc20170.1	Putative GDP-L-fucose synthase 2 (AtGER2)
**TRINITY_DN116626_c0_g2_i1**	−1.78	0.04	44.55	27.16	7.87	0	0	0	CHR25	Acc28491.1	L10-interacting MYB domain-containing protein (probable)
**TRINITY_DN122105_c0_g1_i1**	−1.37	0.01	187.5	146.22	121.01	57.2	46.43	45.44	CHR25	Acc28707.1	Ubiquinol oxidase 1a, mitochondrial, Precursor (putative)
**TRINITY_DN126144_c0_g3_i6**	−1.83	0.02	34.34	17.76	15.74	0	0	0	CHR25	Acc29051.1	COBW domain-containing protein 1 (COBP) (probable)
**TRINITY_DN117186_c0_g2_i2**	−1.82	0.02	65.9	38.64	38.37	10.04	4.94	1.82	CHR25	Acc29489.1	UPF0162 protein PD_0709 (probable)
**TRINITY_DN128356_c0_g1_i6**	−1.47	0.02	107.67	206.8	122.98	53.19	39.51	30.9	CHR25	Acc29080.1	Pectin acetylesterase 8, Precursor (putative)
**TRINITY_DN113632_c0_g1_i1**	−1.44	0.03	451.11	1051.75	476.19	107.38	263.75	209.04	CHR25	Acc12497.1	BURP domain protein RD22, Precursor (similar to)
**TRINITY_DN130185_c0_g1_i4**	−2.3	2.26 × 10^−5^	47.34	67.89	82.64	4.01	8.89	5.45	CHR29	Acc33009.1	CRM-domain containing factor CFM3, chloroplastic/mitochondrial (ZmCFM3), Precursor

**Table 2:** Putative identities and molecular formulae of metabolites exhibiting difference at *p* < 0.05 based on untargeted metabolomics. The samples comprised a total of N = 130 leaf samples (no technical replication) from diploid backcross AI247 and AJ247 vines with (N = 65) or without (N = 65) the *A. eriantha* allele for marker KCH00062.

Column	RT (min)	Putative Candidate(s)	Molecular Weight	Formula	Group Area: Eriantha Allele (+)	Group Area: Eriantha Allele (−)	Ratio: +/−	Log2Fold Change	*p*-Value
C18	3.37		312.09	C14 H16 O8	7738.38	3601.05	2.15	1.1	0.0
C18	3.1	caffeoyl quinide	336.08	C16 H16 O8	3969.41	1585.49	2.5	1.32	0.01
C18	1.09		338.06	C15 H14 O9	4282.86	2079.64	2.06	1.04	0.0
C18	2.85		366.13	C13 H22 N2 O10	3257.78	1545.54	2.11	1.08	0.02
C18	4.59	carbohydrate derivative	416.21	C21 H28 N4 O5	90,832.77	16,046.98	5.66	2.5	0.01
C18	4.48	carbohydrate derivative	417.09	C13 H21 N7 O3 P2 S	3259.7	781.94	4.17	2.06	0.0
C18	4.76	carbohydrate derivative	430.22	C22 H30 N4 O5	121,431.57	44,149.79	2.75	1.46	0.01
C18	4.59	carbohydrate derivative	430.22	C22 H30 N4 O5	13,720.41	5974.26	2.3	1.2	0.04
C18	4.08		436.19	C19 H28 N6 O4 S	4271.37	1198.71	3.56	1.83	0.01
C18	4.93	Kaempferol-3-*O*-glucoside	448.1	C21 H20 O11	52,765.53	8300.52	6.36	2.67	0.0
C18	4.35	carbohydrate derivative	456.19	C19 H28 N4 O9	2884.91	1385.24	2.08	1.06	0.03
C18	3.49	spermidine derivative	456.2	C17 H29 N8 O5 P	7872.29	2542.55	3.1	1.63	0.01
C18	4.59	carbohydrate derivative	476.23	C19 H28 N10 O5	9879.76	4216.6	2.34	1.23	0.02
C18	4.76	carbohydrate derivative	476.23	C16 H37 N4 O10 P	100,274.28	36,739.65	2.73	1.45	0.01
C18	4.99	Isorhamnetin 3-galactoside	478.11	C22 H22 O12	46,274.3	2049.91	22.57	4.5	0.04
C18	3.49	Fatty acid like	491.24	C23 H33 N5 O7	12,980.96	3197.7	4.06	2.02	0.02
C18	3.55	organic acid	498.21	C23 H36 N2 O6 P2	11,612.98	4222.33	2.75	1.46	0.0
C18	4.37	carbohydrate derivative	516.15	C23 H24 N4 O10	13,026.27	3798.38	3.43	1.78	0.02
C18	3.85		531.22	C25 H44 N O3 P3 S	4708.15	1379.95	3.41	1.77	0.0
C18	3.82	Quercetin-carbohydrate derivative	549.23	C21 H32 N11 O5 P	51,922.7	13,083.19	3.97	1.99	0.01
C18	5.09	glutathione derivative	549.24	C24 H37 N7 O4 P2	22,422.9	6422.03	3.49	1.8	0.02
C18	4.12		561.23	C20 H37 N9 O6 P2	8968.49	2888.64	3.11	1.63	0.03
C18	3.91		565.13	C28 H30 N3 O2 P3 S	3647.66	1762.66	2.07	1.05	0.0
C18	3.06		581.17	C27 H27 N5 O10	3067.62	774.45	3.96	1.99	0.01
C18	3.96	organic acid	586.23	C27 H35 N6 O7 P	6628.24	1273.95	5.2	2.38	0.03
C18	4.65	Luteolin-like	742.38	C36 H57 N8 O3 P3	40,140.01	18,953.97	2.12	1.08	0.03
C18	4.33	glucose derivative	760.39	C38 H56 N4 O12	11,611.09	4014.93	2.89	1.53	0.05
C18	3.16		771.31	C28 H60 N3 O13 P3 S	20,076.74	9413.9	2.13	1.09	0.01
Helic	1.09	Organic acid derivative	145.95		303,336.81	106,554.16	2.85	1.51	0.02
Helic	1.64	carbohydrate derivative	192.08	C11 H12 O3	47,978.16	20,483.51	2.34	1.23	5.34 × 10^−6^
Helic	1.34	glycosylated phenylpropanoid	222.09	C12 H14 O4	33,395.63	11,436.95	2.92	1.55	0.02
Helic	3.54		241.98	C4 H9 N2 O4 P3	51,090.99	21,734.49	2.35	1.23	0.04
Helic	3.53		247.97	C5 H12 O3 S4	29,426.88	4357.26	6.75	2.76	0.01
Helic	1.34		266.08	C13 H14 O6	26,764.49	10,639.77	2.52	1.33	0.03
Helic	5.9	coumaric acid deriv	282.07	C13 H14 O7	9,722,985.89	4,295,374.34	2.26	1.18	0.05
Helic	2.0	coumarin glycoside	324.08	C16 H12 N4 O4	37,094.1	12,640.85	2.93	1.55	1.97 × 10^−5^
Helic	6.99		449.04	C21 H12 N3 O7 P	8,334,381.62	2,709,273.63	3.08	1.62	0.05
Helic	6.21		534.16	C21 H31 N2 O12 P	3488.47	1650.29	2.11	1.08	0.04
Helic	3.35		549.13	C24 H28 N3 O8 P S	13,522.12	4106.46	3.29	1.72	0.0

**Table 3:** Summaries of sequencing pool phenotypes.

Pool ID	Description	Mean AsA mg/100 g FW	SD	Mean Fruit Weight g	SD
1	High AsA/High Fruit weight	385.9	59.5	96.6	10.85
2	High AsA/Low Fruit weight	433.55	57.1	56.6	13.51
3	Low AsA/High Fruit weight	100.15	41.12	95.65	15.13
4	Low AsA/Low Fruit weight	87.25	24.56	59.85	15.04

**Table 4:** Primer sets used for high-resolution melting (HRM) in this study and their target intervals on genome references.

Primer Set Name	Forward Primer	Reverse Primer	Target Interval (*A. eriantha* ‘White’)	Target Interval (*A. chinensis* Red5)
KCH00062	GTGGCATTACTTTCCATATTGGG	TGGGCATTGAGTTGTAACCC	CHR26:8460956-8461055	CHR26:7836781-7836880
CHR26:8193148	AGGATAGTTGGCAATTTCCAGG	TGGTAAGCCCAATAGACTATACCC	CHR26:8898197-8898278	CHR26:8206006-820608
CHR26:8874229	ACATACCATTCGGAAGCGTG	ACTGTAGGAACTGAATAGTGATCG	CHR26:9597461-9597577	CHR26:8887032-8887148
CHR26:8453577	GATAATGCGCCCACAGTTCC	GTTGAACTTTGAAGGAAACCTGC	Not determined	CHR26:8466420-8466503

## Supplementary Materials

The following are available online at https://zenodo.org/: normalised read counts, variant data files and coordinates of homeo-SNPs between *A. chinensis* and *A. eriantha* on the draft ‘Red5’ *A. chinensis* 10.5281/zenodo.3257749; pseudomolecules and annotations for the draft ‘Red5’ *A. chinensis* assembly doi: 10.5281/zenodo.1297304; scaffold files of the low-coverage *A. eriantha* assembly of genotype EA01_01 doi:10.5281/zenodo.1309031; Further details of bioinformatics of the bioinformatics methods are available from the corresponding author on request. The following are available online at https://www.mdpi.com/2223-7747/8/7/237/s1: Table S1 Genome locations, read counts and associated annotations of de novo assembled transcripts mapping to kiwifruit genome assembly Red5_PS1_1.69.0 (https://www.ncbi.nlm.nih.gov/assembly/GCA_003024255.1/) which exhibited differential expression between high and low AsA pools at FDR *p* < 0.05. Table S2 Trinity de novo assembly transcripts related to mannose metabolism that did not exhibit significant expression between AsA pools. Transcipts annotated as GDP-L-fucose synthase include the differentially expressed TRINITY_DN123292_c0_g2_i2.

## Appendix A

**Table A1:** Positions on the ‘Red5’ version PS1.68.5 assembly and allele counts of SNP loci exhibiting significant association with fruit AsA level by CMH tests with adjusted *p*-value < 10^−6^. Allele counts at SNP loci were normalised by resampling to 40 reads and are in the format A:T:C:G:N:del. B denotes Ewens homozygosity information [60].

Chr	Pos	Ref	Pool 1 Counts	Pool 1 B	Pool 2 Counts	Pool 2 B	Pool 3 Counts	Pool 3 B	Pool4 Counts	Pool 4 B	Padj	Odds Ratio	Gene Model
CHR26	703957	A	45:59:0:0:0:0	0.3	45:52:0:0:0:0	0.3	70:14:0:0:0:0	0.2	59:0:0:0:0:0	0	1.77 × 10^−10^	12.4	
CHR26	703959	T	0:47:59:0:0:0	0.3	0:49:52:0:0:0	0.3	0:70:14:0:0:0	0.2	0:59:0:0:0:0	0	6.89 × 10^−10^	11.8	
CHR26	703960	T	54:52:0:0:0:0	0.3	49:50:0:0:0:0	0.3	12:72:0:0:0:0	0.18	0:59:0:0:0:0	0	6.07 × 10^−9^	11.8	
CHR26	703962	T	0:46:63:0:0:0	0.3	0:49:53:0:0:0	0.3	0:71:14:0:0:0	0.19	0:59:0:0:0:0	0	1.63 × 10^−10^	12.8	
CHR26	704022	G	64:0:0:58:0:0	0.3	42:0:0:45:0:0	0.3	11:0:0:65:0:0	0.18	0:0:0:38:0:0	0	8.99 × 10^−7^	10.5	
CHR26	704029	T	64:54:0:0:0:0	0.3	40:42:0:0:0:0	0.3	11:65:0:0:0:0	0.18	0:43:0:0:0:0	0	1.33 × 10^−7^	11.5	
CHR26	955933	T	0:19:22:0:0:0	0.3	0:17:18:0:0:0	0.3	0:40:2:0:0:0	0.08	0:57:0:0:0:0	0	3.44 × 10^−7^	47.5	Acc29265
CHR26	2453803	C	0:14:20:0:0:0	0.29	0:20:19:0:0:0	0.3	0:0:50:0:0:0	0	0:0:44:0:0:0	0	2.13 × 10^−6^	Inf	Acc29296
CHR26	2453811	A	13:0:14:0:0:0	0.3	21:0:18:0:0:0	0.3	49:0:0:0:0:0	0	45:0:0:0:0:0	0	8.99 × 10^−7^	Inf	
CHR26	3001514	G	19:0:0:12:0:0	0.29	15:0:0:10:0:0	0.29	0:0:0:34:0:0	0	0:0:0:27:0:0	0	5.0 × 10^−6^	Inf	
CHR26	5096282	A	38:24:0:0:0:0	0.29	45:22:0:0:0:0	0.28	68:2:0:0:0:0	0.06	76:0:0:0:0:0	0	8.99 × 10^−7^	41.8	Acc29359
CHR26	5136827	A	80:0:0:35:0:0	0.27	69:0:0:36:0:0	0.28	82:0:0:8:0:0	0.13	104:0:0:0:0:0	0	2.72 × 10^−6^	10.2	
CHR26	6431391	A	18:0:0:15:0:0	0.3	16:0:0:23:0:0	0.29	39:0:0:0:0:0	0	37:0:0:0:0:0	0	2.74 × 10^−6^	Inf	
CHR26	6431400	A	17:0:0:13:0:0	0.3	12:0:0:23:0:0	0.28	37:0:0:0:0:0	0	41:0:0:0:0:0	0	5.39 × 10^−7^	Inf	
CHR26	6432735	G	15:0:0:12:0:0	0.3	21:0:0:15:0:0	0.3	2:0:0:40:0:0	0.08	0:0:0:37:0:0	0	3.28 × 10^−6^	55.6	
CHR26	6433469	G	33:0:0:32:0:0	0.3	21:0:0:49:0:0	0.27	2:0:0:48:0:0	0.07	0:0:0:74:0:0	0	1.19 × 10^−6^	44.1	
CHR26	6516587	C	0:22:19:0:0:0	0.3	0:29:15:0:0:0	0.28	0:4:44:0:0:0	0.13	0:0:39:0:0:0	0	2.03 × 10^−7^	28.7	
CHR26	7647158	T	0:12:13:0:0:0	0.3	0:19:18:0:0:0	0.3	0:43:0:0:0:0	0	0:45:0:0:0:0	0	1.69 × 10^−6^	Inf	Acc29482
CHR26	7647167	T	0:12:13:0:0:0	0.3	0:22:20:0:0:0	0.3	0:38:0:0:0:0	0	0:45:0:0:0:0	0	4.35 × 10^−6^	Inf	Acc29482
CHR26	8214875	T	0:39:0:0:0:0	0	0:37:6:0:0:0	0.18	0:16:21:0:0:0	0.3	0:18:30:0:0:0	0.29	4.92 × 10^−6^	19.4	Acc29512
CHR26	8414723	C	11:0:16:0:0:0	0.29	20:0:14:0:0:0	0.29	0:0:40:0:0:0	0	0:0:40:0:0:0	0	5.77 × 10^−6^	Inf	Acc29527
CHR26	8571426	G	20:0:0:12:0:0	0.29	17:0:0:14:0:0	0.3	0:0:0:34:0:0	0	0:0:0:33:0:0	0	1.73 × 10^−6^	Inf	Acc29540
CHR26	9081448	G	0:16:0:13:0:0	0.3	0:16:0:22:0:0	0.3	0:1:0:46:0:0	0.05	0:0:0:47:0:0	0	3.28 × 10^−6^	108.3	
CHR26	9263130	G	18:0:0:18:0:0	0.3	14:0:0:18:0:0	0.3	39:0:0:0:0:0	0	35:0:0:0:0:0	0	4.2 × 10^−6^	Inf	
CHR26	9436328	A	18:0:0:18:0:0	0.3	12:0:0:28:0:0	0.27	32:0:0:2:0:0	0.1	43:0:0:0:0:0	0	2.18 × 10^−7^	44.2	Acc29619

## Appendix B. Untargeted Metabolomics Analysis Methodology

### Appendix B.1. LC-MS Data Acquisition

#### Appendix B.1.1. LCMS System

The system consisted of a Thermo Scientific™ (San Jose, CA, USA) Q Exactive™ Plus Orbitrap coupled with a Vanquish™ UHPLC system (Binary Pump H, Split Sampler HT, Dual Oven). Calibrations were performed immediately prior to sample analysis batch with Thermo™ premixed solutions (Pierce™ LTQ ESI Positive and negative ion calibration solutions, catalogue numbers: 88322 and 88324 respectively).

#### Appendix B.1.2. Aqueous Normal Phase Conditions

A 2 μL aliquot of each prepared extract was separated with a mobile phase consisting of 0.1% formic acid in acetonitrile (A) and 5 mM ammonium acetate in water (B) by normal phase chromatography (hypersil Gold HILIC 1.9 µm, 100 mm × 2.1 mm, P/N:26502-102130) maintained at 55 °C with a flow rate of 400 µL/min. A gradient was applied: 0–1 min/5%B, linear increase to 12 min/98%B, isocratic 16 min/98%B, equilibration 16–17 min/5%B, isocratic to end 20 min/5%B.

#### Appendix B.1.3. Reverse Phase Conditions

A 2 μL aliquot of each prepared extract was separated with a mobile phase consisting of 0.1% formic acid in type 1 water (A) and 0.1% formic acid in acetonitrile (B) by reverse phase chromatography (Accucore Vanquish C18 1.5 µm, 100 mm × 2.1 mm, P/N: 27101-102130, Thermo Scientific) maintained at 40 °C with a flow rate of 400 µL/min. A gradient was applied: 0–1 min/0%B, linear increase to 7 min/50%B, linear increase to 8 min/98%B, isocratic to 11 min/98%B, equilibration 11–12 min/0%B, isocratic to end 17 min/0%B.

The eluent from (H) and (C18) chromatography was scanned from 0.5–16 and 0.4–11.5 min respectively by API-MS (Orbitrap) with heated electrospray ionisation (HESI) at 350 °C in the negative and positive mode with capillary temperature of 320 °C. Data were acquired for precursor masses from *m*/*z* 80–1200 amu (H) and *m*/*z* 100–1500(C18) at 70 K resolution (AGC target 3 × 10^6^, maximum IT 100 ms, profile mode) with data dependent ms/ms for product ions generated by normalised collision energy (NCE: 35, 45, 65) at 17.5 K resolution (TopN 10, AGC target 2 × 10^5^, Maximum IT 50 ms, Isolation 1.4 *m*/*z*).

Samples were grouped based on family and KHC00062 marker genotype, and additionally each was subsampled to create a sample mix of each group and solution blanks. Samples were analysed by four analytical methods (two columns, aqueous reverse phase (C18) and aqueous normal phase (Helic) with two ionisation modes -, + (n or p)) creating four datasets (Cn, Cp, Hn, Hp).

### Appendix B.2. Data Processing

Data were processed with the aid of Xcalibur^®^4.1 and Compound Discoverer 3.0 (Berlin/Heidelberg, Germany, Waltham, MA, USA). Calculated exact molecular weights generated from *m*/*z* ions and spectra features (isotope ratios, precursor and product fragment ions) were utilised to predict compound formula, targeted search lists, internal and published library spectra and known compounds/metabolites from selected parameters. Differential analysis was applied based on grouping sets to filter compounds of interest. Significant features were manually interpreted or confirmed with reference to theoretical spectra features and or literature from SciFinder™ with chemistry associated with keywords or in combination with chemical classes/structure search of interest.
